# Supply-Side Barriers in Accessing Human Papillomavirus Screening for Cervical Cancer Prevention in Rural India: Evidence From a Cross-Sectional Study

**DOI:** 10.7759/cureus.73145

**Published:** 2024-11-06

**Authors:** Shyamkumar Sriram

**Affiliations:** 1 Rehabilitation and Health Services, University of North Texas, Denton, USA

**Keywords:** cervical cancer, cervical cancer screening, data management, healthcare infrastructure, hpv screening, infection prevention, personnel training, policy adherence, rural india, supply chain

## Abstract

Background: Cervical cancer is a leading cause of cancer-related mortality among women worldwide, particularly in low- and middle-income countries. In India, rural areas face a disproportionate burden of cervical cancer cases. Effective screening methods, such as Human Papillomavirus (HPV) testing, are recommended by the World Health Organization (WHO) for early detection and prevention. However, the uptake of these screening methods in rural India is significantly low due to various supply-side barriers. This study aims to evaluate the readiness and capacity of PHCs in rural India to conduct HPV screening tests.

Materials and methods: This cross-sectional study in Pondicherry, India, examined cervical cancer screening and management at 15 primary health centers (PHCs) through a structured questionnaire. It assessed personnel training for HPV screening, availability of screening tests, healthcare infrastructure functionality, supply chain efficiency, infection prevention practices, medicine and diagnostic supply availability, referral protocols, data management practices, policy adherence, and equipment availability.

Results: Visual inspection with acetic acid was available in all PHCs, while more advanced tests like cytology, HPV testing, loop electrosurgical excision procedure (LEEP), colposcopy, and histology/pathology were generally absent. Only one PHC had personnel trained for cytology processing, with none trained for other advanced procedures or HPV testing. The study identified significant gaps in healthcare infrastructure, trained personnel, and supply chain logistics.

Conclusion: Enhancing cervical cancer screening and treatment in PHCs necessitates investments in infrastructure, training, and data systems. Key priorities include upgrading equipment like colposcopes and implementing a robust Health Management Information System (HMIS). Collaboration with stakeholders is essential for effective resource allocation and capacity building.

## Introduction

Cervical cancer remains a leading cause of cancer-related mortality among women globally, particularly in low- and middle-income countries [1]. In India, cervical cancer accounts for a significant proportion of cancer cases, with rural areas bearing a disproportionate burden [2]. According to the Indian Council of Medical Research's National Cancer Registry Program (ICMR-NCRP), the estimated number of cervical cancer cases in the country in 2023 was more than 3.4 lakh [3]. The Human Papillomavirus (HPV) is a well-established etiological agent in the development of cervical cancer, making HPV screening a crucial preventive measure. The World Health Organization (WHO) recommends HPV testing as the preferred method for cervical cancer screening due to its high sensitivity [4]. Despite the availability of effective screening techniques, the uptake of HPV screening in rural India is markedly low. This disparity can largely be attributed to various supply-side barriers that impede access to these vital services [5].

Supply-side barriers encompass the limitations within the healthcare system that hinder the provision and accessibility of medical services. These barriers are multifaceted and include inadequate healthcare infrastructure, a shortage of trained healthcare personnel, unreliable supply chains for medical equipment and supplies, and inefficient health system management [6]. In the context of rural India, these barriers are further compounded by geographic, socio-economic, and cultural factors, making it challenging to implement and sustain effective HPV screening programs [7,8].

The healthcare infrastructure in rural India is often characterized by under-resourced facilities, which lack the essential equipment and resources to conduct HPV screenings. Primary health centers (PHCs) and community health centers (CHCs), which form the backbone of rural healthcare, frequently operate without the necessary diagnostic tools and laboratory capabilities to perform and process HPV tests [8-10]. This infrastructural deficit significantly limits the availability and reach of screening services, leaving a large segment of the population without access to early detection measures [11].

The availability of skilled healthcare providers is crucial for the successful implementation of HPV screening programs. However, rural India faces a critical shortage of trained gynecologists, pathologists, and healthcare workers capable of administering and interpreting HPV tests [12,13]. This shortage is exacerbated by the maldistribution of healthcare professionals, who are predominantly concentrated in urban centers, leaving rural areas underserved. Moreover, the lack of ongoing training and professional development opportunities for healthcare workers in rural settings further hampers their ability to effectively conduct and manage screening programs [13-15].

Effective HPV screening relies on a consistent supply of testing kits and other medical supplies. However, rural India struggles with significant logistical challenges that disrupt the supply chain. Poor road infrastructure, limited transportation options, and bureaucratic hurdles often result in delays and stockouts of essential supplies. These supply chain issues are further aggravated by financial constraints, making it difficult to maintain a steady and reliable flow of the necessary materials for screening [16].

The overall management of the health system plays a pivotal role in the successful implementation of HPV screening programs [17,18]. In rural India, systemic issues such as fragmented service delivery, lack of coordination between different levels of care, and inadequate health information systems create substantial barriers. These issues lead to poor record-keeping, difficulties in tracking screening coverage, and challenges in ensuring follow-up care for individuals who test positive for HPV. Effective management practices are essential to streamline processes, enhance coordination, and improve the overall efficiency of HPV screening programs [17-19].

This study aims to comprehensively evaluate the readiness and capacity of PHCs in rural India to conduct HPV screening tests. The objectives include identifying the types of cervical cancer screening tests and assessing the availability of trained personnel capable of providing these screenings. Furthermore, the study will examine the infrastructure, supply chain mechanisms, and data management practices supporting HPV screening and analyze the infection prevention practices in place related to these screenings.

## Materials and methods

This cross-sectional study was conducted at 15 PHCs spread across Pondicherry, India, including locations such as Karayambuthur, Bahour, Kirumampakkam, Thavalakuppam, Ariyankuppam, Reddiyarpalayam, Karikalampakkam, Ariyur, Koodapakkam, Abishegapakkam, Villianur, Gorimedu, Mettupalayam, Nettapakkam, and Sedarapet.

The data collection for this study was done in collaboration with Aaru Padai Veedu Medical College, Pondicherry, through a research agreement with Ohio University, with all required permissions obtained from the District Medical Office in Pondicherry, India. The study was carried out at the designated PHCs in the region. 

The questionnaire used in this cross-sectional study addressed key aspects crucial to its objectives. It evaluated the availability and training status of healthcare personnel qualified to conduct HPV screening tests and administer cervical cancer screenings at 15 PHCs in Pondicherry, India. This assessment was complemented by a detailed inquiry into the types and accessibility of cervical cancer screening tests offered at each PHC, providing insights into diagnostic capabilities.

Study design and duration

This cross-sectional study was conducted over the course of one year, from 2023 to 2024, at PHCs located across Pondicherry, India.

Inclusion and exclusion criteria for PHCs

PHCs were selected based on specific inclusion criteria: they had to be government-operated, serve rural communities, and actively offer cervical cancer screening services. PHCs that did not have an established cervical cancer screening program or lacked adequate staff to participate were excluded from the study.

Development and validation of the questionnaire

The questionnaire was developed through a comprehensive review of existing literature, focusing on key areas such as infrastructure readiness, staff competencies, and the availability of cervical cancer screening tools from “The World Health Organization's (WHO) Improving Data for Decision-making toolkit, a resource for cervical cancer prevention and control programs”. To ensure validity, it was reviewed by public health and oncology experts and then piloted at two PHCs not included in the final sample. The feedback from this pilot phase helped refine the questionnaire, ensuring clarity and reliability. The final version demonstrated strong internal consistency, with a Cronbach’s alpha score of 0.7.

Data collection and respondents

Data were collected through questionnaires completed by PHC staff and doctors involved in the cervical cancer screening programs. Respondents were chosen based on their involvement in the screening process, with the questionnaires typically filled out by senior staff members or physicians responsible for overseeing the screening services.

Infrastructure and supply chain management were rigorously assessed to gauge the adequacy and functionality of healthcare facilities, ensuring efficient delivery of medical supplies and equipment necessary for cervical cancer screening and treatment. The study also focused on infection prevention practices and protocols implemented at the PHCs to safeguard patient safety and hygiene standards.

Moreover, the availability and accessibility of critical medicines and diagnostic supplies were scrutinized to understand the PHCs' readiness to handle cervical cancer cases. Protocols governing patient referrals to higher-level healthcare facilities were reviewed to ensure seamless continuity of care.

Data management practices were documented to ensure the accuracy, confidentiality, and compliance of data collection, storage, and management with regulatory standards. Additionally, the study assessed PHCs' adherence to national and international healthcare policies and guidelines relevant to cervical cancer prevention and management, providing a comprehensive view of policy implementation. Lastly, an inventory of essential medical equipment necessary for effective cervical cancer screening and diagnostic procedures was conducted, contributing valuable insights into the overall healthcare delivery capabilities across the studied PHCs in Pondicherry.

Institutional ethical approval was obtained from the Institutional Review Board of Ohio University (IRB project 23-E-101). Ohio University’s Institutional Review Board deemed it exempt from review since no interventions were carried out.

## Results

Table 1 shows that PHCs offer various cervical cancer screening tests. Cytology (sample collection) is available in one PHC, while cytology (processing), HPV testing (sample collection and processing), visual inspection with Lugol’s iodine (VILI), loop electrosurgical excision procedure (LEEP), colposcopy, endocervical curettage, and histology/pathology are not available in any PHCs. Visual inspection with acetic acid (VIA) is available in 14 PHCs.

**Table 1:** Assessment of infrastructure facilities available in PHCs for cervical cancer screening

Services	No of PHCs
Cytology (sample collection)	1
Cytology (processing)	0
HPV test (sample collection)	0
HPV test (processing)	0
Visual inspection with acetic acid (VIA)	14
Visual inspection with Lugol’s iodine (VILI)	0
Loop electrosurgical excision procedure (LEEP)	0
Colposcopy	0
Endocervical curettage	0
Histology/pathology	0

 Table 2 shows only one PHC has personnel available to be trained for cytology sample collection.

**Table 2:** Assessment of personnel readiness for training in cervical cancer screening tests in PHCs

Potential staffing	No of PHCs
Cytology (sample collection)	1
Cytology (processing)	0
HPV test (sample collection)	0
HPV test (processing)	0
Visual inspection with acetic acid (VIA)	0
Visual inspection with Lugol’s iodine (VILI)	0
Loop electrosurgical excision procedure (LEEP)	0
Colposcopy	0
Endocervical curettage	0
Histology/pathology	0
Other	0

Table 3 shows only one PHC has staff to perform cytology processing and VIA.

**Table 3:** Evaluation of personnel availability for cervical cancer screening tests in PHCs

Staffing	No of PHCs
Cytology (sample collection)	0
Cytology (processing)	1
HPV test (sample collection)	0
HPV test (processing)	0
Visual inspection with acetic acid (VIA)	1
Visual inspection with Lugol’s iodine (VILI)	0
Loop electrosurgical excision procedure (LEEP)	0
Colposcopy	0
Endocervical curettage	0
Histology/pathology	0
Other	0

Table 4 shows that all 15 PHCs have essential infrastructure facilities, including clean examination rooms with privacy options, handwashing stations, client-accessible washrooms, reliable electrical power, areas for confidential counseling, communication equipment (e.g., phones), and storage space for instruments.

**Table 4:** Evaluation of infrastructure facilities in PHCs for enhanced cervical cancer screening services

Infrastructure	No of PHCs
Physical layout and space: Functional, clean and uncluttered private examination room (or large room with privacy screens)	15
Hand Wash area (sink with running water/bucket with spigot; soap)	15
Washroom/bathroom for client use	15
Reliable electrical power (Note: may be considered not essential for some services)	15
Space for confident counseling	15
Communication equipment (e.g., phone)	15
Storage space for instruments	15

Table 5 shows that the procurement and supply chain processes involve regular assessments of equipment and supplies at all 15 PHCs. Preventive measures are in place to avoid stockouts, ensuring each PHC is well-equipped. Supplies arrive consistently on time, with a streamlined reordering process integrated into routine operations.

**Table 5:** Assessment of procurement and supply chain availability for cervical cancer screening in PHCs

Procurement and supply chain	No of PHCs
Regular assessment of equipment and supply levels	15
Prevention and management of stock out	15
Supplies arrive in a predictable amount of time when ordered	15
Reordering of supplies is routine (e.g., incorporated in regular workflow with designated roles and ordering schedules)	15

Table 6 shows that all 15 PHCs are equipped with examination tables, clean examination gloves, and clocks or timers. Metal specula, sponge/ring forceps, and a bright white light source are available in 13 PHCs. Additionally, all PHCs receive a 3%-5% acetic acid solution and have access to clean cotton balls or swabs in large quantities.

**Table 6:** Evaluation of equipment and supplies available for cervical cancer screening in PHCs

Equipment and supplies	No of PHCs
Examination tables	15
Instrument trays/trolleys or similar surfaces	15
Metal specula (in the screening clinic)	13
Sponge/ring forceps or wooden orange or kebab sticks	13
Gallipots/other small dishes	13
Clean examination gloves	15
Bright white light source	13
Clock/watch/timer	15
Clean cotton balls/cotton swabs – large	15
3%–5% acetic acid	15

Table 7 shows that the criteria for equipment and supplies at the PHCs ensure essential needs are met. All 15 PHCs are equipped with liquid soap or alcohol-based hand sanitizer, buckets for collecting contaminated instruments, and a 0.5% chlorine solution. Each PHC can sterilize instruments using a functional autoclave or 2%-4% glutaraldehyde, with containers provided for storage.

**Table 7:** Assessment of availability of infection and prevention items in PHCs

Criteria	No of PHCs
Liquid soap for hands or alcohol-based hand sanitizer	15
Buckets for collection of contaminated instruments and for instrument processing	15
0.5% Chlorine solution	15
Ability to sterilize and store properly (check the method(s) that apply): Functional autoclave, or 2%–4% glutaraldehyde (including sterile water to rinse) Note: Need only one of the above methods to meet the standard AND containers to store sterilized instruments	15
Ability to high-level disinfect (HLD) and store properly (check all that apply): Pressure cooker for steam-based high-level disinfection Sufficient gas to run pressure cooker burner 2%–4% glutaraldehyde (including sterile or boiled water to rinse) 70%–90% ethyl or isopropyl alcohol (for cryotherapy tips only) Note: Need only one of the above methods to meet the standard AND Containers to store HLD instruments	14
Normal and hazardous waste bags and baskets	15
Ability to properly dispose of hazardous wastes (e.g., incinerator or burial pit)	3

For high-level disinfection (HLD), each PHC can use a pressure cooker for steam disinfection, 2%-4% glutaraldehyde, or 70%-90% alcohol for cryotherapy tips, although only 14 have appropriate storage containers. All 15 PHCs receive normal and hazardous waste bags, but only three can properly dispose of hazardous waste via incineration or burial.

Table 8 shows that the criteria for medical supplies across PHCs include a variety of essential items. All 15 PHCs are stocked with pain relief medicines, such as Panadol and Ibuprofen, as well as antibiotics for the treatment of cervicitis and sexually transmitted infections (STIs) according to national guidelines. HIV test kits and pregnancy testing supplies are also available at all 15 PHCs. However, none of the PHCs currently have HPV specimen collection tubes or test kits and cartridges (e.g., GeneXpert).

**Table 8:** Assessment of availability of medicines and testing in PHCs

Criteria	No of PHCs
Pain relief medicines (e.g. Panadol, Ibuprofen, other)	15
Antibiotics for treatment of cervicitis and sexually transmitted infections (STIs) per national guidelines	15
HPV specimen collection tubes and/or test kits and cartridges (e.g. GeneXpert)	0
HIV test kits	15
Pregnancy testing	15

Table 9 shows that all 15 PHCs have identified their referral sites, and referral mechanisms, including the flow of information and how results are obtained by the client and referring provider/facility, are clearly described. Additionally, the results of referrals are consistently documented, and facility staff assess and attempt to address barriers to referral in all 15 PHCs. Fourteen PHCs have referral guidelines and readily available referral forms.

**Table 9:** Assessment of referral mechanisms in PHCs

Criteria	No of PHCs
Referral sites for the facility are identified	15
Referral guidelines are available	14
Referral forms are readily available	14
Referral mechanisms are described (flow of information and how results are obtained by client and referring provider/facility)	15
Results of the referrals are documented	15
Facility staff assess and attempt to address barriers to referral	15

Table 10 shows that currently, none of the PHCs have the latest blank client screening or monthly summary forms available. Fourteen PHCs possess the most recent registers or logbooks, while nine PHCs maintain client privacy through secure data management practices. However, there is no Health Management Information System (HMIS) for reporting cervical cancer screening and treatment in any of the PHCs. Additionally, there are no designated staff or schedules in place to facilitate data entry or review of results.

**Table 10:** Assessment of data management in 15 PHCs

Criteria	No of PHCs
Latest version of blank client screening/treatment forms (if used) and monthly summary forms available	0
Latest version of the register or logbooks available	14
Data management/storage ensures the privacy of client information	9
Health management information system (HMIS) for reporting cervical cancer screening and treatment	0
Designated staff and schedule to ensure reporting data accessible to providers for data entry and/or reviewing results	0

Table 11 shows that policies and guidelines at the PHCs are well-maintained, with 14 out of 15 PHCs displaying or providing easy access to up-to-date national guidelines. These include organized binders or folders containing cervical cancer prevention and control guidelines, screening and treatment policies, and Infection Prevention and Control (IPC) protocols.

**Table 11:** Assessment of policies and guidelines across 15 PHCs

Policies and guidelines	No of PHCs
Relevant and current national guidelines and policies are displayed or readily available in a proper binder or folder (e.g. national cervical cancer prevention and control programed guidelines; other policies and guidelines related to screening and treatment offered at the facility; Infection Prevention and Control (IPC) guidelines).	14

Table 12 shows that PHCs have basic equipment for handling and processing specimens, including tube racks and sample processing machinery. However, none of the PHCs have essential items like pipettes, 4°C refrigerators, surge protectors, or temperature and humidity sensors.

**Table 12:** Non-consumable equipment and supplies for HPV testing: a comprehensive overview

Criteria	Minimum requirement		Availability in number of PHCs
Tube rack or other mechanism for storing and transporting specimens in a vertical position	At least 1	1	
Machinery for processing samples and power cables	1	1	
Fixed volume pipette	1	0	
Variable volume repeater pipette	1	0	
4°C refrigerator	1	0	
Surge protector (minimum 1500 VA)	1	0	
Temperature and humidity sensor	1	0	

Table 13 shows that none of the PHCs have the essential supplies and equipment needed for sample collection and processing. These include sample collection brushes or swabs, sample transport medium, assay microplates and reagents (HPV test kits), safety glasses, laboratory coats, non-powder gloves, micropipette tips, tube racks, and paper towels. All of these items are unavailable across the PHCs.

**Table 13:** Assessment of consumable equipment and supplies for HPV testing

Materials	No of PHCs
Sample collection brush or swab	0
Sample transport medium	0
Assay microplate and reagents (HPV test kit)	0
Safety glasses	0
Laboratory coat	0
Non-powder gloves	0
Micropipette tips	0
Tube racks	0
Paper towels	0

Table 14 shows that none of the PHCs have critical equipment such as a light microscope, colposcope, biopsy forceps, ring forceps, endocervical curette, or LEEP electrosurgical tools (including generator, smoke evacuator, electrodes, and electrosurgery pen). However, at least one PHC has coated, non-conducting speculums with tubing, ring/sponge-holding forceps, long tissue forceps, blood pressure machines/cuffs, and long needle holders. These items are essential for conducting comprehensive cervical cancer screening and treatment procedures.

**Table 14:** An overview of non-consumable equipment and supplies – other services LEEP - Loop electrosurgical excision procedure

Equipment	No of PHCs
Cytology	
Light microscope	0
Colposcopy, biopsy, endocervical curettage	
Colposcope	0
Biopsy forceps	0
Ring forceps	0
Endocervical curette	0
LEEP	
LEEP electrosurgical generator (with smoke evacuator) and electrode handle	
Return electrode	0
Loop and ball electrodes	0
Dispersive plate/pad	0
Electrosurgery pen	0
Coated, non-conducting speculum, speculum tubing	1
Ring/sponge-holding forceps	1
Long tissue forceps	1
Blood pressure machine/cuff	1
Long needle holder	1

Table 15 shows that the availability of essential equipment in PHCs is limited. For cytology, only one PHC has glass slides, cover slips, a spatula or brush, cytology fixative, and liquid transport medium, while specimen labels are unavailable across all centers. Lugol’s iodine for VILI is absent in all PHCs. For colposcopy, biopsy, and endocervical curettage, Monsel’s paste, and formalin specimen bottles are also not available. Additionally, key supplies for LEEP procedures, such as spinal needles, lignocaine, and formalin bottles, are missing across all PHCs.

**Table 15:** Inventory of essential consumables for comprehensive healthcare in PHCs VILI - Visual inspection with Lugol’s iodine, LEEP - Loop electrosurgical excision procedure, PHC - Primary Health Centers

Supplies	No of PHCs
Cytology	
Glass slides and cover slips	1
Spatula or brush	1
Cytology fixative	1
Liquid transport medium in individual specimen containers	1
Specimen labels (and marker/pencil for labeling)	0
VILI	
Lugol’s iodine	0
Colposcopy, biopsy, endocervical curettage	
Monsel’s paste	0
Specimen bottles with 10% formalin	0
LEEP	
22-, 25-, or 27-gauge spinal needle, 3.5 inches long	0
1%–2% lignocaine with 1:200,000 epinephrine	0
1%–2% lignocaine plain	0
Monsel’s paste	0
Specimen bottles with 10% formalin	0

Table 16 shows that all PHCs consistently provide essential equipment for gynecological examinations and procedures. This includes Graves metal bivalve specula in medium and large sizes, ring/sponge-holding forceps, kidney dishes, and gynecological examination tables. Additionally, PHCs are equipped with Macintosh or rubber sheets, goose-neck lamps or other reliable light sources, instrument trays or trolleys, and specimen cups for vinegar. Movable and adjustable stools, timers or clocks, privacy screens, and sheets and gowns are also available. This standardized provision of equipment ensures comprehensive facilities for gynecological care across all 14 PHCs.

**Table 16:** Critical non-consumables and equipment for via in primary health centers

Equipment	No of PHCs
Specula – Graves, metal bivalve specula (medium and large)	14
Ring/sponge-holding forceps	14
Kidney dishes	14
Gynecological examination table	14
Macintosh or rubber sheet	14
Goose-neck lamp (or other good light source such as torchlight)	14
Instrument trays or trolleys	14
Specimen cups (vinegar)	14
Movable and adjustable stool	14
Timer, clock, or watch	14
Privacy screens	14
Sheets and gowns	14

Table 17 shows that all PHCs consistently provide the following essential supplies: clean, non-sterile examination gloves (box of 100), 3%-5% acetic acid (white vinegar in a 1 L bottle), rolls of cotton wool for making cotton balls, wooden kebab sticks, small cotton swabs, and non-sterile gauze rolls. Additionally, they stock batteries (size AA), chlorine for preparing a 0.5% solution, condoms for retracting lax vaginal walls, and tongue depressors for the same purpose. This uniform availability across all 14 PHCs supports standardized practices for gynecological care and procedures.

**Table 17:** VIA equipment and consumables overview VIA - Visual inspection with acetic acid

Equipment	No of PHCs	
Clean, non-sterile examination gloves – box of 100		14
3%–5% acetic acid (white vinegar) – 1 L bottle		14
Roll of cotton wool to make cotton balls		14
Wooden kebab sticks		14
Small cotton swabs		14
Non-sterile gauze roll		14
Batteries (size AA)		14
Chlorine to make 0.5% solution		14
Condoms to retract vaginal walls that are lax		14
Tongue depressors to retract vaginal walls that are lax		14

## Discussion

The analysis reveals that while PHCs show adequacy in certain areas, significant gaps persist in resources and services related to cervical cancer screening and treatment. Studies, like Tapera et al., emphasize the need for effective and comprehensive planning in cervical cancer screening programs, particularly in low-resource settings, advocating for substantial investments and stressing the crucial role of governments in mobilizing necessary resources. Petersen et al. and Dhillon et al. highlight considerable variability in facility readiness for cervical cancer screening across different states and tiers of India's public healthcare system. They identify infrastructure and staffing as major barriers at PHCs, essential for referring high-risk patients, and underline the importance of district-level policy focus to enhance screening efforts [5,20-22].

Availability of screening tests and personnel: Regarding screening tests and personnel availability, while VIA is universally present in 14 PHCs, other tests like cytology, HPV testing, LEEP, colposcopy, and histopathology are lacking across most PHCs, severely limiting effective cervical cancer detection and management. Trained personnel for these tests are also scarce, with only one PHC having staff trained in cytology processing and none equipped for advanced procedures or HPV testing.

Studies by Onyenwenyi et al., Sneha et al., and Dsouza et al. stress the importance of training PHC nurses and workers in visual inspection methods, suggesting integration of these cost-effective techniques into training curricula to enhance rural cervical cancer prevention efforts [23,24]. Srivastava et al. discuss various effective screening techniques applicable in low-resource settings, including visual inspection methods and cytology, proven to reduce cervical cancer incidence. Zhetpisbayeva et al. emphasize the need for targeted measures to increase cervical cancer screening uptake in rural populations, addressing higher prevalence rates. Sriram et al. propose planning strategies tailored to sociodemographic and community-level factors to improve local HPV prevention efforts [8,9,22].

Infrastructure and equipment

All PHCs in Pondicherry meet basic infrastructure standards according to Indian Public Health Standards (IPHS), providing essential facilities such as private examination rooms, handwashing areas, and communication equipment. They also maintain adequate stocks of essential supplies like examination tables, instrument trays, gloves, and acetic acid, as per IPHS guidelines. However, there is a significant deficiency in critical equipment necessary for advanced procedures, such as colposcopes, biopsy forceps, and HPV testing kits, which limits the comprehensive care these PHCs can provide [21].

Data management and policies

Standardized data management systems, like HMIS, are absent in all PHCs, impeding effective tracking and reporting of screening and treatment outcomes - an essential requirement of IPHS standards. Ensuring that policies and guidelines in most PHCs are readily accessible and consistently updated is vital for maintaining quality standards in cervical cancer prevention and control [21,25,26].

Studies, like Bhatla et al., advocate for scaling up cervical cancer prevention through widespread HPV vaccination and primary HPV testing as integral components of standard care, highlighting their potential to markedly reduce cervical cancer rates. Dhillon et al. underline significant barriers posed by infrastructure and staffing deficits at PHC levels to effective implementation of cervical cancer screening guidelines, emphasizing the need for targeted interventions to enhance screening outcomes. Meanwhile, Mustafa et al. and Dsouza et al. offer practical insights into healthcare system challenges and recommend systemic improvements to bolster capacities for cervical cancer prevention and management, aiming to ensure equitable access to screening and treatment services [18,21,25-27].

Limitations

This study has several limitations that should be acknowledged. First, the cross-sectional design only provides a single point-in-time assessment, limiting the ability to observe changes in infrastructure, training, or resource availability at the PHCs over time. Second, as the study was conducted solely in PHCs in Pondicherry, the generalizability of the findings to other regions with differing healthcare systems may be limited. Third, the use of self-reported data from healthcare staff introduces the possibility of reporting bias, as respondents may have either overestimated or underestimated their facility’s resources and challenges. Moreover, while the questionnaire focused on key structural and procedural factors, it may have overlooked other important dimensions, such as patient experiences and adherence to follow-up care. Finally, this study did not assess long-term outcomes related to cervical cancer screening and treatment, such as patient survival rates or the effectiveness of specific interventions. Future studies should consider longitudinal designs to capture the long-term impact of improvements in infrastructure and personnel training on screening and treatment outcomes.

## Conclusions

Addressing these deficiencies through targeted investments in infrastructure, equipment, personnel training, and data management systems is crucial for improving cervical cancer screening and treatment outcomes in PHCs in Pondicherry and similar settings worldwide. Prioritizing the training of healthcare personnel in cytology processing and advanced screening techniques, alongside securing funding for essential equipment such as colposcopes and HPV testing kits, is essential. Implementing a robust HMIS tailored for cervical cancer screening and treatment will streamline data entry, reporting, and analysis, thereby enhancing program monitoring and evaluation. Ensuring adherence to and regular updates of national guidelines and protocols across all PHCs will uphold standardized practices and ensure quality care delivery. By systematically addressing these challenges, PHCs can significantly enhance their capacity to deliver comprehensive cervical cancer screening and treatment services. Collaborating with government health departments, non-governmental organizations, and international health organizations will facilitate resource mobilization and capacity building in underserved areas. This integrated approach is pivotal for reducing the burden of cervical cancer through early detection and effective management.

## Appendices

Health facility questionnaire

Improving data for decision-making: a toolkit for cervical cancer prevention and control programs (who.int)

https://www.who.int/publications/i/item/9789241514255
